# Psychological Determinants of Medication Adherence in Stroke Survivors: a Systematic Review of Observational Studies

**DOI:** 10.1007/s12160-017-9906-0

**Published:** 2017-04-18

**Authors:** Elise Crayton, Marion Fahey, Mark Ashworth, Sarah Jane Besser, John Weinman, Alison J. Wright

**Affiliations:** 1 Department of Primary Care and Public Health Sciences, Division of Health and Social Care Research, Faculty of Life Sciences & Medicine, King’s College London, 6th Floor, Addison House, Guy’s Campus, London, SE1 1UL UK; 2 King’s College London, Institute of Pharmaceutical Sciences, London, UK

**Keywords:** Systematic review, Stroke, Medication adherence, Psychological determinants

## Abstract

**Background:**

Medications targeting stroke risk factors have shown good efficacy, yet adherence is suboptimal. To improve adherence, its determinants must be understood. To date, no systematic review has mapped identified determinants into the Theoretical Domains Framework (TDF) in order to establish a more complete understanding of medication adherence.

**Purpose:**

The aim of this study was to identify psychological determinants that most influence stroke survivors’ medication adherence.

**Methods:**

In line with the prospectively registered protocol (PROSPERO CRD42015016222), five electronic databases were searched (1953–2015). Hand searches of included full text references were undertaken. Two reviewers conducted screening, data extraction and quality assessment. Determinants were mapped into the TDF.

**Results:**

Of 32,825 articles, 12 fulfilled selection criteria (*N* = 43,984 stroke survivors). Tested determinants mapped into 8/14 TDF domains. Studies were too heterogeneous for meta-analysis. Three TDF domains appeared most influential. Negative emotions (‘Emotions’ domain) such as anxiety and concerns about medications (‘Beliefs about Consequences’ domain) were associated with reduced adherence. Increased adherence was associated with better knowledge of medications (‘Knowledge’ domain) and stronger beliefs about medication necessity (‘Beliefs about Consequences’ domain). Study quality varied, often lacking information on sample size calculations.

**Conclusions:**

This review provides foundations for evidence-based intervention design by establishing psychological determinants most influential in stroke survivors’ medication adherence. Six TDF domains do not appear to have been tested, possibly representing gaps in research design. Future research should standardise and clearly report determinant and medication adherence measurement to facilitate meta-analysis. The range of determinants explored should be broadened to enable more complete understanding of stroke survivors’ medication adherence.

**Electronic supplementary material:**

The online version of this article (doi:10.1007/s12160-017-9906-0) contains supplementary material, which is available to authorized users.

## Introduction

Stroke is the second leading cause of death in developed countries [1] and can lead to life-altering consequences [2]. Guidelines recommend the use of medication for secondary prevention of stroke [3–5]. These medications target stroke risk factors such as high blood pressure and high serum cholesterol values. The medications prescribed for stroke risk factor control have shown good efficacy in the literature and reductions in the rate of stroke recurrence per annum [6, 7], with cumulative reductions in relative risk by as much as 75% [8]. Nonetheless, adherence rates to stroke prevention medications remain suboptimal [e.g. 9, 10].

For the purpose of this review, medication adherence is defined as “the extent to which the patient's action matches the agreed recommendations” [10]. Among individuals with long-term conditions, 33–50% of patients were non-adherent to long-term medications [10]. Among stroke survivors, a recent systematic review reported a pooled prevalent non-adherence rate of 30.9% (95% CI 26.8–35.3%) [11]. A better understanding of the underlying reasons for suboptimal adherence will enable more informed intervention development. Therefore, the aim of this systematic review was to identify psychological determinants that influence medication adherence in stroke survivors.

Current evidence has considered the role of psychological, demographic, system, biological and other factors when trying to understand medication adherence. Determinants, such as beliefs about medication, presence of other comorbid conditions, age and lack of clinical symptoms have been previously identified as influential in stroke survivors’ medication adherence [12–15]. The negative consequences of taking medications, including unpleasant side effects and drug interactions, as well as difficulty accessing the prescribing clinician or pharmacy and issues with prescription costs, could also contribute to non-adherence [e.g. 11, 16–18]. Moreover, current interventions have had limited success at effectively improving medication adherence [e.g. 19]. Some determinants of medication adherence, such as age, gender or stroke type [11], are not easily modified. Therefore, a better understanding of the modifiable determinants of medication adherence is required to facilitate the design of behaviour change interventions. Psychological determinants, defined as determinants of, or relating to, the mind or mental processes, also relating to or affecting a person’s emotional state [20], are one type of potentially modifiable determinant. Considerable research effort has been made to link psychological determinants to the behaviour change techniques (BCTs) likely to change each one [21, 22]. This could facilitate adherence intervention design. Consequently, the current review focused on identifying the strongest psychological determinants of medication adherence in stroke survivors and considered the quality of the primary studies.

Many theories of the psychological influences on behaviour have been developed (e.g. Theory of Planned Behaviour [23] and Health Belief Model [24, 25]). However, such theories of health behaviour have been subject to a number of criticisms, including not always operationalising the constructs clearly, not considering the context in which a behaviour occurs and an over emphasis on rational, deliberative determinants. As there is considerable unexplained variance in adherence, the addition of further predictor variables should enhance the theories (see [26]). In partial response to the latter two criticisms, the Theoretical Domains Framework (TDF) has been developed [27, 28].

The TDF was developed via an expert consensus approach. Behaviour change professionals identified constructs from many major behaviour change theories. The identified constructs were clustered using open and closed sort tasks, grouping similar constructs together to form, what the authors termed, a domain. After revisions, 14 key domains were established (Knowledge; Skills; Social/Professional role and identity; Beliefs about capabilities; Optimism; Beliefs about consequences; Reinforcement; Intentions; Goals; Memory, Attention and Decision processes; Environmental context and resources; Social influences; Emotions; and Behavioural regulation [28]). The TDF provides more comprehensive coverage of influences on behaviour than any single theory of behaviour and was therefore used as a theoretical framework in this review. A further advantage of the TDF is that the domains can be mapped to BCTs that are thought to be most likely to change each type of determinant [28, 29].

The aim of this systematic review was to identify psychological determinants that influence medication adherence in stroke survivors. The secondary aim was to establish the magnitude of the relationships between the psychological determinants and stroke survivors’ medication adherence. To our knowledge, there has not yet been a review, which has mapped identified determinants into the TDF in order to establish a more complete understanding of medication adherence in stroke survivors.

## Methods

This review includes studies focused on people with a clinical diagnosis of stroke (ischaemic or haemorrhagic) and prescribed medications that targeted stroke risk factors for secondary prevention. The Preferred Reporting Items for Systematic Reviews and Meta-Analyses (PRISMA) guidelines were followed [30]. The systematic review protocol was prospectively registered on PROSPERO (CRD42015016222).

### Search Strategy and Selection Process

**Fig. 1:**
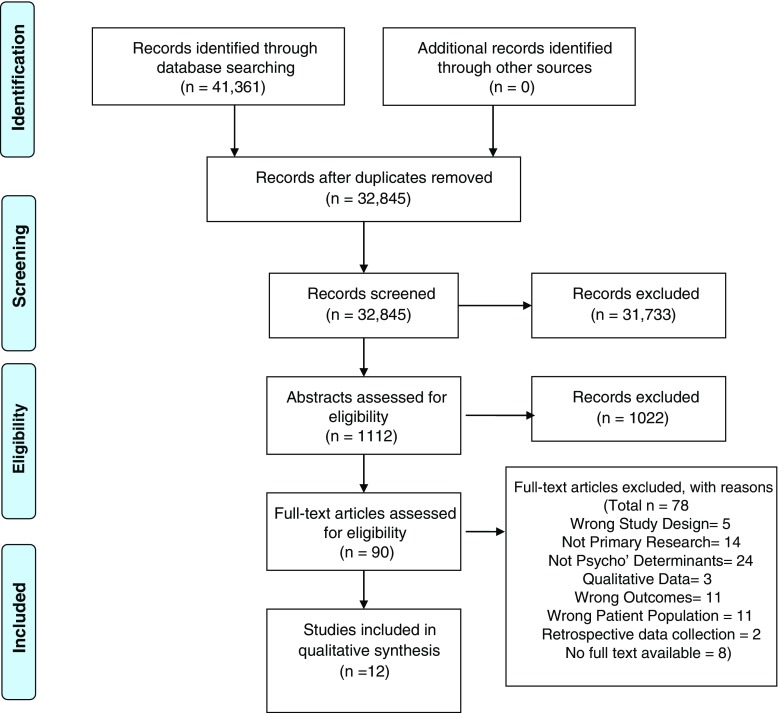
PRISMA diagram

The search targeted literature investigating psychological determinants of medication adherence in stroke survivors. A multi-method search was undertaken using combined terms for stroke AND adherence AND psychological determinants and a combination of subject heading and free text searching where applicable (See Supplement 1 for tailored search strategy). Sources included MEDLINE, EMBASE, PsycINFO, CINAHL, Web of Science (inclusive of conference proceedings) and reference lists of included full text articles. The search was limited to English language as this was the only fluent language understood by the review team. The inception date of the search was 1953 because literature regarding “compliance” in healthcare started to appear from the early 1950s [31]. Eligibility and selection of relevant articles were assessed by first conducting title/abstract review and then by assessing full texts according to predefined inclusion/exclusion criteria. COVIDENCE software was used to manage this process. The selection process, data extraction and quality assessment were performed independently by two reviewers (EC, MF). A third reviewer resolved conflicts and cross-checked data extraction (AJW). Reviewer EC extracted data from all included full texts. Reviewer MF extracted data from a proportion (10%) of the full texts and extracted all subjective and outcome data from the remaining texts (90%). If reviewers required more information, the authors were contacted. Seven of the 19 authors contacted responded. Figure 1 displays the PRISMA diagram of the search and selection process.

### Criteria for Study Inclusion and Exclusion

Inclusion criteria:

- Studies with a sample of stroke survivors or mixed transient ischaemic attack (TIA)/stroke survivors who were ≥18 years of age and had been prescribed medication(s) that targeted at least one stroke risk factor
- Primary research studies with quantitative research designs measuring at least one psychological determinant and medication adherence

Exclusion criteria:

- Studies with a sample of stroke survivors <18 years of age
- Mixed condition samples where stroke only data could not be obtained
- Reviews (systematic, narrative or meta-analytic), studies applying retrospective data collection and qualitative study designs

Randomised control trials (RCTs) were not explicitly excluded from the search strategy, but only one RCT identified was relevant to the review research question [32]. The RCT had been informed and was a sequel to an observational study identifying psychological determinants [13]. Therefore, for this review, the inclusion of the observational study design was considered most appropriate.

### Data Extraction and Analysis

#### Data Extraction

Data extraction was completed using a proforma developed for this review, in accordance with Cochrane guidance [33]. The data extracted included: (1) participant clinical and demographic characteristics, (2) study design and methods, (3) adherence measures, (4) identified psychological determinants and (5) statistical information.

#### Analysis

Summary data from each full text were extracted. The analysis within this review focused on the effect sizes of the relationship between medication adherence/persistence and the determinants given. Data collection methods from the included papers were too heterogeneous to allow for a meta-analysis. The determinants were grouped into the relevant TDF domains. To identify which domains were most influential to adherence, assessment of the domains with a higher number of tested determinants with significant associations was carried out. The number of papers and samples that a determinant was tested in was also extracted and used to establish domains with the most influence on medication adherence. Domains were considered more influential when a larger proportion of the tested determinants had significant associations with adherence and where significant associations were found in a higher proportion of the samples in which at least one determinant from the domain was tested.

#### Quality Assessment

Quality assessment was conducted independently by two reviewers (EC, MF) using the 13-item checklist designed by Walburn and colleagues [34] to appraise studies of attitudes to medicines. The checklist assesses items such as a priori aims, definition/size of population under investigation, sample size calculations and justification that the sample is representative of population. The checklist is not intended to provide a defined cutoff study quality score, below which studies should be excluded from analysis. Instead, using the checklist facilitated qualitative consideration of the impact of study design features on findings.

#### Determinant Mapping

Two coders (EC and SJB), with qualifications in Health Psychology (MSc, PhD and MSc), independently mapped the identified psychological determinants into TDF domains. Domain definitions were taken from the most recent version of the TDF at the time of this review [28]. One coder (MA), a qualified general practitioner with experience in mental health research, resolved disputes. Determinants were coded into the most suitable domain, or domains if it was agreed that the determinant fitted into more than one, or not coded if none of the domains seemed appropriate. Where possible, the wording of the items used to measure a determinant was checked to ensure domains were coded in line with what was measured, rather than simply the label given to a determinant by the study authors. Cohen’s kappa for agreement between the two coders [35] was *k* = 0.69 (SE = 0.07 [95% CI = 0.56–0.82]), indicating substantial agreement.

## Results

A search from inception until November 2015 produced a total of 32,845 articles (duplicates removed). Titles and abstracts were screened, producing 90 full texts to assess. Following assessment of full texts, 12 papers reporting on seven samples met inclusion criteria (Fig. 1).

### Study Characteristics

Detailed study characteristics can be found in Table 1. The 12 papers were derived from seven samples, with another two of the papers posing a potential for overlap. Therefore, results will now be considered by displaying the number of papers (x/12) and number of samples (x/7), relevant to each factor. Most studies (9/12, 5/7) assessed medication adherence. Three of the twelve studies (2/7 samples) assessed medication persistence [40, 43, 44]. The total sample size was 43,984 (range 25 to 21,077). Research was conducted in four countries (USA, Australia, Sweden and UK) across three continents. Settings for participant recruitment included hospital (5/12, 4/7) [39–41, 43, 44], community (6/12, 2/7) [36–38, 42, 45, 46] and an outpatient setting (1/12, 1/7) [13]. The reported stroke subtypes included ischaemic (6/12, 5/7) [13, 39–41, 43, 44], haemorrhagic (3/12, 3/7) [39, 40, 42] and TIA (6/12, 2/7) [37, 38, 43–46], with the majority of papers reporting samples with mixed subtypes (75%). In seven papers (3/7 samples), the stroke subtype was either undefined or only some of the sample’s stroke subtypes were defined.

**Table 1:** Summary of each included full text article

Author/country	Design	Participants	Number	Medication adherence measure	Psychological determinants	Psychological determinant measure	Key findings [95% CI]	*P* values	% of max quality score^a^
Sample 1									
Bushnell (2010) / USA [43]	Prospective	Ischaemic stroke (1712) and TIA (465)	2177	Comparison- discharge vs. current medications (measured by modified MMAQ)	Understanding how to refill medications	Unclear from paper	OR 1.64 [1.04–2.58]	*P* = 0.03	61.5
					Understanding why medications are being taken	Unclear from paper	OR 1.81 [1.19–2.76]	*P* = 0.006	
					EQ-5D score	EuroQoL-5D	OR 2.33 [1.24–4.38]	*P* = 0.009	
Bushnell (2011) / USA [44]	Prospective	Ischaemic stroke and TIA	2092	Comparison- discharge vs. current medications (measured by modified MMQ)	Receiving medication instructions	The Primary Care Assessment Survey	OR 1.43 [1.13–1.81]	*P* < 0.003	61.5
					Understanding medication side effects	Unclear from paper	OR 1.29 [1.02–1.63]	*P* = 0.032	
					Understanding why medications are being taken	Unclear from paper	OR 1.49 [1.03–2.17]	*P* = 0.036	
Sample 2									
Edmondson (2013)/ USA [45]	Cross-sectional	TIA and undefined stroke	535	8 item MMAQ	PTSD symptoms	PCL-S	OR 1.02 [1.00–1.05]	0.1 > *p* > 0.05^c^	90.9
					Specific concerns	BMQ (specific)	OR 1.17 [1.10–1.25]	*p* < 0.05^c^	
					Depressive symptoms	PHQ-8	OR 1.02 [0.97–1.08]	*p* > 0.05^c^	
Kronish (2012)/ USA [36]	Cross-sectional	Undefined stroke	535	8 item MMAQ	Likely PTSD	PCL-S	OR 2.69 [1.71–4.23]	*P* < 0.05^c^	90.9
					Possible PTSD	PCL-S	OR 1.86 [1.27–2.74]	*p* < 0.05^c^	
					Depressive symptoms	PHQ-8	OR 1.12 [0.88–1.42]	*p* > 0.05^c^	
Kronish (2013) /USA [46]	Cross-sectional	TIA and undefined stroke	600	8 item MMAQ	High concerns about medications	Modified BMQ Specific Concerns (X4 items)	OR 5.09 [2.81–9.24]	*p* < 0.001	90.9
					Low perceived need of medications	Modified BMQ Specific Necessity	OR 1.23 [0.79–1.91]	*P* = 0.36	
					Low knowledge of stroke risk factors	NV-Qx1 State 3 most important things would recommend to others to lower stroke risk	OR 1.22 [0.76–1.96]	*P* = 0.42	
					Low trust in personal doctor	Adapted Trust in Doctors Scale (×3 items)	OR 1.30 [0.84–2.01]	*P* = 0.23	
					Perceive discrimination due to race, ethnicity, education, or income	NV-5 point Likert scale	OR 1.79 [1.14–2.81]	*P* = 0.01	
Phillips (2014) / USA [37]	Cross-sectional	TIA (284) and undefined stroke (316)	600	8 item MMAQ	Necessity beliefs	Adapted BMQ Specific	*β* = 0.25 [0.07–0.42]	*P* < 0.01	72.7
					Concerns	Adapted BMQ Specific	*β* = −0.81 [−0.96 to −0.66]	*P* < 0.001	
Phillips (2015) / USA [38]	Cross-sectional	TIA (284) and undefined stroke (316)	600	8 item MMAQ	Affective illness items	NV-Q ×1 Level of worry about future stroke	*r* = −.27 *p* = 0.001 *β* −0.14, *R* ^2^ 0.02 (F (1, 564) = 12.33)	*P* < 0.001	72.7
					Cognitive illness items	NV-Q ×2 How well blood pressure and cholesterol is controlled	*r* = 0.29 *p* = 0.001 *β* 0.18, *R* ^2^ 0.03 (F(1, 564) = 22.16)	*P* < 0.001	
					Affective treatment items	BMQ Specific Concerns (×3 items)	*r* = −0.40 *p* = 0.001 *β* −0.31, *R* ^2^ 0.08 (F(1, 564) = 56.71)	*P* < 0.001	
					Cognitive treatment items	BMQ-specific necessity (×3 items) + NV-“‘How much do you think medicines can help prevent strokes?”	*r* = 0.12 *p* = 0.01 *β* 0.13, *R* ^2^ 0.02 (F(1, 564) = 11.62)	*P* < 0.01	
Sample 3									
Coetzee (2008) /Australia [39]	Prospective	Ischaemic (14) and haemorrhagic (11) stroke	25	Q1 and 2 on TAS Pill Counts	(Partner) Emotional dyscontrol	EFQ	*r* = −0.66	*P* < 0.01	84.6
					Language skills	EFQ	*r* = −0.44	*P* < 0.001	
					Memory	EFQ	*r* = −0.54	*P* < 0.001	
					Planning and organisation	EFQ	*r* = −0.52	*P* < 0.001	
					Anger	ESDQ	*r* = −0.63	*P* < 0.01	
					Emotional dyscontrol	ESDQ	*r* = −0.76	*P* < 0.001	
					Helplessness	ESDQ	r = −o.64	*P* < 0.01	
					Inertia	ESDQ	*r* = −0.61	*P* < 0.01	
					Fatigue	ESDQ	*r* = −0.52	*P* < 0.01	
					Indifference	ESDQ	*r* = −0.54	*P* < 0.01	
					EUPHORIA	ESDQ	*r* = −0.51	*P* < 0.01	
					(Partner) Emotional dyscontrol	ESDQ	*r* = −0.51	*P* < 0.001	
					(Partner) Inertia	ESDQ	*r* = −0.51	*P* < 0.01	
					Anxiety	HADS	*r* = −0.74	*P* < 0.001	
					Specific necessity	BMQ Specific	*r* = −0.53	*P* < 0.01	
					Care received at home	Questions in TAS	*r* = 0.53	*P* < 0.01	
Sample 4									
O’Carroll (2011) /UK [13]	Prospective	Ischaemic stroke	180	Urine samples MARS	Specific medication concerns	BMQ Specific	*β* = −0.254	*P* < 0.01	76.9
					MMSE score	MMSE	*β* = 0.000 *NS*	*p* > 0.05^c^	
					Perceived benefit of medication	NV-Adapted Q.s^d^	*β* = 0.273	*P* < 0.001	
					RMBT score	RBMT	*β* = 0.167 *NS*	*p* > 0.05^c^	
					Risk perception of further stroke	NV-Visual analogue 0–100 scale	*β* = −0.044 *NS*	*p* > 0.05^c^	
					Illness perceptions-acute/chronic timeline	IPQ	*β* = 0.002 *NS*	*p* > 0.05^c^	
					Illness perceptions- treatment control	IPQ	*β* = −0.021 *NS*	*p* > 0.05^c^	
					Specific necessity	BMQ specific	*β* = −0.022 *NS*	*p* > 0.05^c^	
					Desire for medications now	NV-Adapted Q.s^1^	*β* = −0.140 *NS*	*p* > 0.05^c^	
					HADS total	HADS	*β* = 0.064 *NS*	*p* > 0.05^c^	
Sample 5									
Glader (2010) /Sweden^b^[40]	Prospective	Ischaemic and undefined stroke	21,077	Data Linkage- RiksStroke with the Swedish Prescribed Drug Register	Support of next of kin	Items from the RiksStroke Register	AH: OR 1.13 [1.02–1.25] S: *NS* AP: *NS* W: OR 0.98 [0.76–1.26]	*p* ≤ 0.001 *p* > 0.05^c^ *p* > 0.05^c^ *P* ≤ 0.05	69.2
					Self-perceived general health	Items from the RiksStroke Register	AH: OR 0.86 [0.76–0.98] S: OR 0.69 [0.59–0.80] AP: OR 0.79 [0.70–0.89] W: *NS*	*P* ≤ 0.02 *P* ≤ 0.001 *P* ≤ 0.001 *p* > 0.05^c^	
					Low mood	Items from the RiksStroke Register	AH: OR 0.88 [0.79–0.98] S: OR 1.12 [0.98–1.28] AP: OR 0.92 [0.83–1.02] W: *NS*	*p* ≤ 0.001 *P* < 0.09 *P* ≤ 0.04 *p* > 0.05^c^	
					Satisfaction with hospital care and support	Items from the RiksStroke Register	AH: *NS* S: *NS* AP: *NS* W: *NS*	*p* > 0.05^c^ *p* > 0.05^c^ *p* > 0.05^c^ *p* > 0.05^c^	
Sample 6									
Sjölander (2011) /Sweden^b^ [41]	Prospective	Ischaemic stroke (men: 9331; women: 9016)	19,347	Prescription refills	Self-reported depression	Items from the RiksStroke Register	Men: PR 0.96 [0.88–1.05] Women: PR 1.00 [0.93–1.08]	*p* > 0.05^c^ *p* > 0.05^c^	69.2
					Self-reported bad general health	Items from the RiksStroke Register	Men: PR 0.99 [0.90–1.09] Women: PR 0.97 [0.89–1.06]	*p* > 0.05^c^ *p* > 0.05^c^	
					Dissatisfied with care	Items from the RiksStroke Register	Men: PR 0.92 [0.74–1.14] Women: PR 0.93 [0.75–1.16]	*p* > 0.05^c^ *p* > 0.05^c^	
					Dissatisfied with support	Items from the RiksStroke Register	Men: PR 0.99 [0.89–1.10] Women: PR 0.99 [0.90–1.10]	*p* > 0.05^c^ *p* > 0.05^c^	
Sample 7									
Sjölander (2013) /Sweden [42]	Cross-sectional	Haemorrhagic (40) and undefined stroke (538)	578	MARS	Specific necessity	BMQ	OR 0.90 [0.83–0.98]	*P* = 0.079	84.6
					Specific concerns	BMQ	OR 1.12 [1.05–1.21]	*P* < 0.001	
					General overuse	BMQ	OR 1.29 [1.14–1.45]	*P* < 0.001	
					General harm	BMQ	OR 1.12 [1.01–1.24]	*P* = 0.038	
					General benefit	BMQ	OR 0.77 [0.68–0.87]	*P* < 0.001	

*NS* not significant, *MMAQ* Morisky Medication Adherence Questionnaire, *MARS* Medication Adherence Report Scale, *TAS* Treatment Assessment Schedule, *BMQ* Beliefs About Medicines Questionnaire, *PCL-S* Modified PTSD Checklist-Specific to stroke/mini stroke, *EQ-5D* EuroQoL-5D, *PHQ-8* 8-item Patient Health Questionnaire Depression Scale, *EFQ* Everyday Functioning Questionnaire, *ESDQ* The Emotional and Social Dysfunction Questionnaire, *HADS* The Hospital Anxiety and Depression Scale, *MMSE* The Mini-Mental State Examination, *RMBT* The Rivermead Behavioural Memory Test, *IPQ* The Illness Perception Questionnaire, *NV* non-validated, *AH* anti-hypertensives, *S* statins, *AP* anti-platelets, *W* warfarin

^a^Percentage of Maximum quality score (see supplementary Material 3 for full quality scoring table)

^b^Potential overlap of samples as same data source was utilised across different dates

^c^Exact *P* values not reported in original paper;

^d^Trewby, P. N., Reddy, A. V., Trewby, C. S., Ashton, V. J., Brennan, G., & Inglis, J. (2002). Are preventive drugs preventive enough? A study of patients’ expectation of benefit from preventive drugs. Clin Med., 2(6), 527–533

Time periods between measurement of determinants and adherence varied, with 6/12 papers, 2/7 samples using cross-sectional designs [36–38, 41, 45, 46] and follow-up time frames for prospective studies of 5–6 weeks (1/12, 1/7) [13], 3 months (2/12, 2/7) [42, 43], 12 months (2/12, 2/7) [39, 44] and 24 months (1/12, 1/7) [40]. A range of questionnaire items (validated and non-validated) was used to measure psychological determinants. Some papers did not clearly describe how determinants were measured.

### Measurement of Adherence

A variety of methods were used to measure medication adherence (Supplement 2). These included the use of self-report measures such as the Medication Adherence Report Scale (MARS) and more objective methods such as conducting pill counts and monitoring prescription refills. In total, seven different methods were applied (3 subjective, 4 objective), either alone or in conjunction with another. Six articles (50%, 5/7 samples) named the specific medications being assessed for adherence. Of these, five considered antiplatelet, anti-hypertensive, cholesterol-lowering and anti-coagulant medications [39–41, 43, 44] and one assessed adherence to antiplatelet, anti-hypertensive and cholesterol-lowering medications [13].

### Quality Assessment

Study quality was varied (Supplement 3). Checklist scores ranged from 8 to 10 (mean = 9.3) out of a possible 13. All included studies reported explicit a priori aims, a sample definition and size, inclusion/exclusion criteria, a response/dropout rate where applicable and whether the research was independent of routine practice. However, only two studies gave a sample size calculation [13, 42]. In addition, although seven studies stated the response/dropout rate [13, 39–44], which ranged from 56 to 96%, only two provided justification for these rates [13, 39]. There was no clear justification of sample representativeness in four studies [13, 39, 43, 44]. In addition, the majority of included studies had designed questionnaires or interview schedules purposely for the research derived from validated and non-validated measures. Three studies did not make the original questionnaire available or provide sufficient information on how all determinants were measured [13, 43, 44], and four studies did not justify the reliability/validity of the measures used [40, 41, 43, 44].

### Determinant Mapping

There were 48 distinct determinants measured across the 12 papers, reporting on seven samples. The most common determinants (6/12 papers, 4/7 samples) were variations of *concerns about medications* and *beliefs about necessity of medications.* Five of 12 articles (4/7 samples) also assessed *depression* as a determinant of medication adherence. Over half the tested determinants were only measured in one study. Table 2 displays the identified determinants from the review mapped into TDF domains. Determinants tested in the papers could be mapped into 8/14 domains. There were no tested determinants that mapped into ‘Social/Professional role and identity’, ‘Optimism’, ‘Reinforcement’, ‘Goals’, ‘Environmental context and resources’ and ‘Behavioural regulation’. One tested determinant, *quality of life* (as measured by increments of 10% in EuroQoL-5D score) could not be mapped into the TDF, as no definition seemed appropriate. Only four determinants (*patient reported* and *partner reported inertia, patient helplessness* and *affective illness items*) were considered to fit within two separate TDF domains (see Table 2 for determinant mapping). All other determinants sat discretely within one domain. A total of 33 distinct determinants, corresponding to seven TDF domains, significantly influenced adherence/persistence behaviour (Table 3). Each domain will now be discussed in turn (for numerical details of observed associations and *p* values, see Table 1).

**Table 2:** Determinants mapped into the theoretical domains framework

Domain	Description^a^	Determinant
Knowledge	*An awareness of the existence of something*	Receiving medication instructions
		Understanding why medications are being taken
		Understanding medication side effects
		Low knowledge of stroke risk factors
		Understanding how to refill meds
		Self-perceived general health
		Self-reported bad general health
Skills	*An ability or proficiency acquired through practice*	Planning and organisation
		Language skills
Social/Professional role and identity	*A coherent set of behaviours and displayed personal qualities of an individual in a social or work setting*	
Beliefs about capabilities	*Acceptance of the truth, reality, or validity about an ability, talent, or facility that a person can put to constructive use*	Cognitive illness items
		Helplessness
Optimism	*The confidence that things will happen for the best or that desired goals will be attained*	
Beliefs about consequences	*Acceptance of the truth, reality, or validity about outcomes of a behaviour in a given situation*	Concerns about medications
		Affective illness items
		Beliefs about necessity
		Perceived benefit of medication
		Cognitive treatment items
		Affective treatment items
		Risk perception of risk of further stroke
		Beliefs about benefit
		Beliefs about overuse
		Beliefs about harm
		Illness perceptions-acute/chronic timeline
		Illness perceptions-treatment control
Reinforcement	*Increasing the probability of a response by arranging a dependent relationship, or contingency, between the response and a given stimulus*	
Intentions	*A conscious decision to perform a behaviour or a resolve to act in a certain way*	Desire for medication now
Goals	*Mental representations of outcomes or end states that an individual wants to achieve*	
Memory, Attention and Decision processes	*The ability to retain information, focus selectively on aspects of the environment and choose between two or more alternatives*	MMSE score
		RMBT score
		Patient memory
Environmental context and resources	*Any circumstance of a person’s situation or environment that discourages or encourages the development of skills and abilities, independence, social competence, and adaptive behaviour*	
Social influences	*Those interpersonal processes that can cause individuals to change their thoughts, feelings, or behaviours*	Support of next of kin
		Low trust in personal doctor
		Perceived discrimination on account of race, ethnicity, education or income
		Dissatisfied with care
		Dissatisfied with support
		Satisfaction with hospital care/support
		Care received at home
		Inertia
		Inertia (rated by partner)
Emotions	*A complex reaction pattern, involving experiential, behavioural, and physiological elements, by which the individual attempts to deal with a personally significant matter or event*	Emotional dyscontrol (rated by partner)
		Emotional dyscontrol
		Anger
		PTSD symptoms
		(Self-reported) Depression/depressive symptoms
		Low mood
		Fatigue
		Indifference
		Euphoria
		Inertia
		Inertia (rated by partner)
		HADS total
		Anxiety
		Helplessness
		Affective Illness Items
Behavioural regulation	*Anything aimed at managing or changing objectively observed or measured actions*	

*PTSD* post-traumatic stress disorder, *HADS* The Hospital Anxiety and Depression Scale, *MMSE* The Mini-Mental State Examination, *RMBT* The Rivermead Behavioural Memory Test

^a^Definitions as stated in Cane et al. 2012 who utilised the definitions from the American Psychological Associations’ Dictionary of Psychology

**Table 3:** Table showing the number of significant determinants (and their negative or positive influence on adherence) within each domain

Domain	Total no. of determinants tested across all papers	No. of determinants significantly associated with better adherence	No. of determinants significantly associated with worse adherence	No. (%) of determinants tested not significantly related to adherence	No. of the 12 papers reporting a test of at least one determinant from this domain	No. of the 7 samples in which at least one determinant from this domain was tested	No. (%) of samples in which determinants were tested and at least one had a significant association with adherence
Knowledge	7	4	1	2 (29%)	5	4	3 (75%)
Skills	2	0	2	0 (0%)	1	1	1 (100%)
Beliefs about capabilities	2	1	1	0 (0%)	2	2	2 (100%)
Beliefs about consequences	12	4	5	3 (25%)	7	4	4 (100%)
Intentions	1	0	0	1 (100%)	1	1	0 (0%)
Memory, attention and decision processes	3	0	1	2 (67%)	2	2	1 (50%)
Social influences	9	2	3	4 (44%)	4	4	3 (75%)
Emotions	15	0	13	2 (13%)	7	4	3 (75%)

#### ‘Knowledge’

Seven distinct determinants mapped into this domain. Two determinants did not have a significant effect on adherence (*self-reported bad general health* and *low knowledge of stroke risk factors).* Five significantly influenced medication adherence/persistence. Generally, greater knowledge was associated with better adherence/persistence. Four significant determinants (*receiving medication instructions, understanding how to refill medications, understanding why medications are being taken* and *understanding medication side effects*) were all related to adherence in this manner. *Self-perceived general health* also had a significant effect on adherence, with poorer *self-perceived general health* associated with poorer medication persistence.

#### ‘Skills’

Two distinct determinants tested (*patient language skills* (reported by a partner) and *patient planning and organisation skills*) mapped to this domain. Both determinants had a significant effect on adherence, with poorer skills associated with worse adherence.

#### ‘Beliefs about Capabilities’

Two distinct determinants were tested, both significantly influencing medication adherence. *Patient helplessness* had a negative impact on adherence. Rating oneself as more helpless was related to poorer adherence. *Cognitive illness items*, assessing patients’ perceived control over stroke risk factors, had a positive impact, with positive responses indicating higher perceived risk factor control related to better self-reported adherence.

#### ‘Beliefs about Consequences’

Twelve distinct determinants were mapped to this domain. Three tested determinants were not found to have a significant effect on medication adherence (*illness perceptions* relating to acute/chronic timelines of a condition, *illness perceptions* referring to treatment control and *perceived risk of further stroke*). Four determinants had a significant positive influence on medication adherence. Greater *perceived necessity of medications* was related to increased adherence (in 2/5 papers). Greater *perceived benefit of medications* (measured in two ways) was related to increased adherence. Higher scores on *cognitive treatment items* (derived from items from the specific necessity subscale of the Beliefs about Medications Questionnaire (BMQ) plus a question regarding how much patients thought their medications could prevent stroke) were related to better self-reported adherence.

Five determinants significantly negatively influenced adherence. When patients had greater *concerns about medications*, *beliefs about medication overuse* and *beliefs about harm from medication* adherence was worse. In addition, worse adherence was related to *affective treatment items*, concerning worries about medications and *affective illness items* concerning worries about stroke.

#### ‘Intentions’

One determinant (*desire for medications now*) was tested, but not found to have a significant effect on adherence.

#### ‘Memory, Attention and Decision Processes’

Three distinct determinants were tested of which two were not significant (*Mini Mental State Exam* (MMSE) *score* and *Rivermead Memory Behavioural Test* (RMBT) *score*). In contrast, *Patient memory* (measured by the Everyday Functioning Questionnaire (EFQ)) significantly influenced medication adherence. Poorer reported memory or memory deficits were related to poorer adherence.

#### ‘Social Influences’

Nine distinct determinants were tested and mapped into this domain. Four (*low trust in personal doctor*, *dissatisfaction with care*, *dissatisfaction with support* and *satisfaction with hospital care/support*) did not have a significant effect on medication adherence/persistence. Two determinants had a significant positive influence on medication adherence/persistence. *Increased support from the next of kin* was related to better persistence with anti-hypertensive and warfarin medications. Moreover, higher levels of *care received at home* were associated with better adherence. In contrast, three determinants negatively influenced adherence. Greater *perceived discrimination due to race, ethnicity, education or income* increased odds of non-adherence. In addition, both *patient-rated* and *partner-rated inertia* influenced adherence negatively. Increasing levels of inertia appeared to relate to increased non-adherence.

#### ‘Emotions’

Fifteen distinct determinants were tested. Two determinants ((*self-reported) depression/depressive symptoms* and Hospital Anxiety and Depression Scale (HADS) *total score*) were not significantly associated with medication adherence. Thirteen determinants had a significant negative influence on adherence/persistence. Adherence/persistence was poorer when patients had greater *patient-reported* or *partner-rated emotional dyscontrol* (measured via two different measures); *post-traumatic stress disorder* (PTSD) *symptoms*; more *anger*; greater *patient-reported* or *partner-rated inertia*; more *fatigue*, *euphoria*, *indifference*, *anxiety*, *low mood*; and higher perceived *helplessness* or scores on *affective illness items* (concerning worries about stroke).

## Discussion

The purpose of this review was to identify psychological determinants that influence medication adherence in stroke survivors. Forty-eight distinct determinants were assessed in 12 articles representing seven samples. The identified determinants were mapped into TDF domains, in order to develop a theoretical understanding of how these determinants influence medication adherence and to inform future work. Based on this review, the ‘Emotions’ (at least one significant determinant in 3/4 samples in which they were tested, 86% of tested associations statistically significant), ‘Knowledge’ (at least one significant determinant in 3/4 samples in which they were tested, 79% of tested associations statistically significant) and ‘Beliefs about consequences’ domains (at least one significant determinant in 4/4 samples in which they were tested, 75% of associations statistically significant) appear to have the strongest influence on medication adherence. The TDF has enabled a holistic approach to understanding medication adherence that will be important in future intervention development.

Within the Emotions domain, emotional distress such as ‘*anxiety*’, ‘*PTSD*’ and ‘*emotional dyscontrol*’ was found to have an influence on medication adherence. Similar findings have emerged in recent literature, corroborating this finding. For example, Gentil and colleagues (2012) assessed anti-hypertensive medication adherence in community-living older adults, finding that adherence was lower when participants had an anxiety or depressive disorder [47]. In addition, a large American study (*n* = 1342) found a significant association between the presence of mental health conditions (anxiety/depression) and difficulty taking anti-hypertensive medications [17].

Within the ‘Knowledge’ domain, *understanding why medications were being taken* and *understanding medication side effects* were found to have influence on medication adherence. Previous literature has found to be similar. A prospective cohort study interviewing 130 stroke survivors and 85 caregivers found large gaps in stroke survivor and caregiver knowledge. For example, 52% of patients were unable to name stroke risk factors. This sample also demonstrated suboptimal health behaviours, with 28% of the patients reporting non-adherence [48]. More recently, a qualitative study identifying barriers to medication adherence with stroke survivors, caregivers and general practitioners in the East of England found similar results [49]. Knowledge of stroke and medication was identified as a patient-level barrier to adherence of secondary prevention medication [49].

Within the ‘Beliefs about consequences’ domain, both *concerns about medication* and *beliefs about the necessity of medication* were the most common determinants with influence. This is commensurate with previous research. In a meta-analytic review assessing the influence of necessity beliefs and concerns on adherence in patients with long-term conditions, higher adherence was related to increased beliefs about necessity of treatment. Likewise, poorer adherence was associated with increased concerns about treatment [50]. Moreover, recent research suggests interventions targeting perceived necessity and concerns about medications increase stroke survivors’ medication adherence [32, 51]. Therefore, those beliefs appear to play a causal role in adherence.

### Quality of Included Studies

All 12 included studies gave clear descriptions of sample demographics, inclusion/exclusion criteria and sample size. Moreover, although there was disparity in the range of sample sizes (25–21,077), there was a pooled sample of 43,984 stroke survivors. These samples were derived from four countries across three continents. In light of this, it can be assumed, with a certain level of confidence, that the reviews findings are generalizable to stroke survivors from developed, western cultures.

There were no defined cutoffs for quality assessment scores. Nevertheless, assessment of the individual items, for each paper, was important to identify gaps in research quality. Only two papers reported a sample size calculation. This is problematic when meta-analysis is not possible, as the finding that some determinants tested did not significantly influence adherence may be due to small sample sizes and underpowered studies, rather than genuine lack of relationships. Moreover, three studies did not make the original questionnaire available or provide sufficient information on measurement of determinants [13, 43, 44]. As several studies used tailor-made questionnaires, including a mix of non-validated and validated scales, it would be helpful to future systematic reviewers to make the full questionnaires available.

Additionally, there were only seven discrete samples of participants across the 12 papers. Two papers [43, 44] reported on the same sample, followed up at different time points. Five papers [36–38, 45, 46] used the same sample of trial participants’ baseline data, with each paper testing different combinations of determinants that might influence medication adherence. We have therefore presented not only the number of determinants tested that were found to be significant but also the proportion of samples in which a type of determinant was both tested and found to be a significant predictor. Given the relatively small number of independent samples included in this review, and the partial coverage of the TDF domains in the included studies, there remains a need for further, well-designed studies of the predictors of medication adherence in stroke survivors.

The secondary aim of this review, to establish the magnitude of the relationships between determinants and behaviour, could not be achieved, as study design choices were too heterogeneous to permit meta-analysis. Measurement of medication adherence was inconsistent across included papers, with different self-report or objective measures chosen, assessing adherence at a number of different time points. This has been identified as an issue in previous research attempting to synthesise data regarding medication adherence [e.g. 19]. All methods of adherence measurement have limitations. Electronic, objective monitoring may be the best currently available option, but nevertheless can be reactive and is costly. Prescription data provides information about medication possession, but not whether medication was taken, while self-report measures are subject to recall and social desirability biases. The majority of studies in the review used self-report measures. Future research might usefully further explore psychological and other predictors of adherence to stroke secondary preventive medication using objective adherence measurement.

It could be suggested that the varying methods of medication adherence measurement add strength to the findings in this review. For example, the determinant *concerns about medications* was measured across five studies (3/7 samples) [13, 37, 42, 45, 46], with a significant relationship identified between this determinant and medication adherence. Across the five studies, medication adherence was measured by three different self-report and one pill count method. Irrespective of the measurement method, a significant relationship was found, thus strengthening the conclusion that there is a relationship between determinant and behaviour.

### Limitations

The number of papers that met final inclusion criteria was small. Authors were contacted (*N* = 19) to request more information or manuscripts relating to data that had previously been presented at conferences or where no full text access could be found, but only seven responded. Other systematic reviewers have reported a similar issue [52].

In spite of the rigorous method applied to determinant mapping, there is still an element of subjectivity in the process. The task relies on interpretation of TDF domain definitions and descriptions of scales provided in the primary studies.

No determinants were mapped into six TDF domains. However, other research has highlighted the importance of some of these domains in sustained behaviour change. For example, Nicholson and colleagues (2014) identified the importance of ‘Environmental context and resources’ with the engagement of physical activity in stroke survivors [53]. The limited breadth of domains tested through this review may represent a ‘file drawer’ problem or limitations in the study designs. This may also be in part due to the inclusion of only psychological determinants, which could be less likely to map into some TDF domains. In particular, the search strategy would have retrieved studies that assessed the association of stroke survivors’ perceptions of their environmental context and resources with adherence, but not studies simply testing whether the presence or absence of different environmental and contextual features influenced adherence. Factors such as prescription costs and health insurance coverage also need to be considered. However, non-adherence remains an issue even in healthcare systems providing universal healthcare coverage and prescriptions free of charge (e.g. 13). Therefore, understanding psychological determinants of adherence remains an important issue to inform intervention design. Despite efforts in the search strategy to access a variety of literature, the 12 selected papers were all identified from the peer-reviewed literature; none were found in the ‘grey’ literature, which could result in publication bias. Future work should aim to measure a broader range of psychological determinants that influence medication adherence in stroke survivors to enhance a more holistic understanding of this behaviour.

## Conclusions

The findings from this review have identified psychological determinants, amenable to change, that influence medication adherence in stroke survivors. ‘Beliefs about Consequences’, ‘Knowledge’ and ‘Emotions’ were the most influential domains. As the TDF underpins the Behaviour Change Wheel, a framework for intervention development, future work can systematically identify the intervention functions and BCTs that target the determinants within each domain. In doing so, there is a greater chance that medication adherence will be enhanced as the intervention will be grounded in both a theoretical understanding of the behaviour and will be applying evidence into practice. Future research should strive for clarity and transparency to support pooling of data, most specifically focused on consistency of medication adherence measurement and testing of a broad range of determinants using standardised measures.

## Electronic supplementary material

ESM 1 (DOCX 34.2 kb)

ESM 2 (DOCX 57.8 kb)

ESM 3 (DOCX 95 kb)
