# Supplementary material for: Psychological Determinants of Medication Adherence in Stroke Survivors: a Systematic Review of Observational Studies
Source: Ann Behav Med. 2017 Apr 18;51(6):833–45. doi: 10.1007/s12160-017-9906-0 (PMC5636868; doi:10.1007/s12160-017-9906-0)
Supplement: Supplementary file 1 — (DOCX 34.2 kb) [file 12160_2017_9906_MOESM1_ESM.docx]

­­­Supplementary Material 1

Tailored Search Strategies

CINAHL (EBSCO interface 1953- November 2015)

| Step Number | Search Terms |
| --- | --- |
| S1 | (MH "Cerebral Ischemia+") OR (MH "Stroke+") |
| S2 | (MH "Intracranial Hemorrhage+") |
| S3 | (MH "Stroke Patients") |
| S4 | TI ( cva* OR stroke* OR poststroke* OR post-stroke* OR post stroke* ) AND AB ( cva* OR stroke* OR poststroke* OR post-stroke* OR post stroke* ) |
| S5 | TI ( cerebrovascular* OR cerebral vascular ) AND AB ( cerebrovascular* OR cerebral vascular ) |
| S6 | TI ( cerebral OR cerebellar OR brain* OR vertebrobasilar ) AND AB ( cerebral OR cerebellar OR brain* OR vertebrobasilar ) |
| S7 | TI ( infarct* OR ischemi* OR ischaemi* OR thrombo* OR apoplexy OR emboli* ) AND AB ( infarct* OR ischemi* OR ischaemi* OR thrombo* OR apoplexy OR emboli* ) |
| S8 | S6 AND S7 |
| S9 | TI ( cerebral OR intracerebral OR intracranial OR brain* OR cerebellar OR subarachnoid ) AND AB ( cerebral OR intracerebral OR intracranial OR brain* OR cerebellar OR subarachnoid ) |
| S10 | TI ( accident* OR hemorrhag* OR haemorrhag* ) AND AB ( accident* OR hemorrhag* OR haemorrhag* ) |
| S11 | S9 AND S10 |
| S12 | S1 OR S2 OR S3 OR S4 OR S5 OR S8 OR S11 |
| S13 | (MH "Medication Compliance") |
| S14 | (MH "Treatment Refusal") OR (MH "Patient Compliance+") OR (MH "Patient Dropouts") |
| S15 | TI ( adhere* OR nonadhere* OR non-adhere* ) AND AB ( adhere* OR nonadhere* OR non-adhere* ) |
| S16 | TI ( complian* OR noncomplian* OR non-complian* ) AND AB ( complian* OR noncomplian* OR non-complian* ) |
| S17 | TI ( (refusal OR refuse*) N3 (medicine* OR medication* OR drug* OR prescription* OR tablet* OR pharmaceutic*) ) AND AB ( (refusal OR refuse*) N3 (medicine* OR medication* OR drug* OR prescription* OR tablet* OR pharmaceutic*) ) |
| S18 | TI ( (medicine* OR medication* OR drug* OR prescription* OR tablet* OR pharmaceutic*) N3 (dropout* OR drop-out* OR drop out*) ) AND AB ( (medicine* OR medication* OR drug* OR prescription* OR tablet* OR pharmaceutic*) N3 (dropout* OR drop-out* OR drop out*) ) |
| S19 | TI ( (medicine* OR medication* OR drug* OR prescription* OR tablet* OR pharmaceutic*) N3 (stop* OR abandon*) ) AND AB ( (medicine* OR medication* OR drug* OR prescription* OR tablet* OR pharmaceutic*) N3 (stop* OR abandon*) ) |
| S20 | TI ( persist* OR concordan* OR accept* OR co-operat* OR cooperat* OR co operat* OR conform* OR medicine taking ) AND AB ( persist* OR concordan* OR accept* OR co-operat* OR cooperat* OR co operat* OR conform* OR medicine taking ) |
| S21 | S13 OR S14 OR S15 OR S16 OR S17 OR S18 OR S19 OR S20 |
| S22 | TI ( knowledg* OR awareness OR understand* OR information ) AND AB ( knowledg* OR awareness OR understand* OR information ) |
| S23 | TI ( skill* OR abilit* OR expertise OR competen* or capabilit* OR response efficacy ) AND AB ( skill* OR abilit* OR expertise OR competen* or capabilit* OR response efficacy ) |
| S24 | TI ( (social N (role or identity)) OR (professional N (role or identity OR confidence)) OR identity ) AND AB ( (social N (role or identity)) OR (professional N (role or identity OR confidence)) OR identity ) |
| S25 | TI ( (belief* N2 capabilit*) OR self-efficacy OR self-confiden* OR perceived competence OR self-esteem OR empowerment OR perceived behavioral control OR perceived behavioural control OR self-control OR self control OR performance ) AND AB ( (belief* N2 capabilit*) OR self-efficacy OR self-confiden* OR perceived competence OR self-esteem OR empowerment OR perceived behavioral control OR perceived behavioural control OR self-control OR self control OR performance ) |
| S26 | TI ( optimis* OR positiv* OR hopefulness OR assurance OR pessimis* OR negativ* ) AND AB ( optimis* OR positiv* OR hopefulness OR assurance OR pessimis* OR negativ* ) |
| S27 | TI ( (belief* N2 (consequence* OR cost*)) OR (outcome N (expectations OR expectancies)) OR risk perception* OR perceived benefit* OR anticipated emotion* OR necessity belief* OR concern* OR response cost* OR coping appraisal OR perceived vulnerability OR perceived likelihood) AND AB ( (belief* N2 (consequence* OR cost*)) OR (outcome N (expectations OR expectancies)) OR risk perception* OR perceived benefit* OR anticipated emotion* OR necessity belief* OR concern* OR response cost* OR coping appraisal OR perceived vulnerability OR perceived likelihood) |
| S28 | TI ( reinforce* OR support OR reward* or incentive* or punish* or consequen* ) AND AB ( reinforce* OR support OR reward* or incentive* or punish* or consequen* ) |
| S29 | TI ( intent* OR intend* OR plan* OR motivation OR health motivation OR pre-contemplation OR contemplation OR preparation OR maintain* or behavior termination OR behaviour termination OR confiden* OR temptation OR consciousness raising OR dramatic relief OR self-reevaluation OR environmental reevaluation OR self-liberation OR helping relationships OR counterconditioning OR reinforcement management OR stimulus control OR social lineation ) AND AS ( intent* OR intend* OR plan* OR motivation OR health motivation OR pre- contemplation OR contemplation OR preparation OR maintain* OR behavior termination OR behaviour termination OR confiden* OR temptation OR consciousness raising OR dramatic relief OR self-reevaluation OR environmental reevaluation OR self-liberation OR helping relationships OR counterconditioning OR reinforcement management OR stimulus control OR social lineation ) |
| S30 | TI ( goal* OR action planning OR target setting ) AND AB ( goal* OR action planning OR target setting ) |
| S31 | TI ( memory OR attention OR decision* OR judgement* OR cogniti* ) AND AB ( memory OR attention OR decision* OR judgement* OR cogniti* ) |
| S32 | TI ( (environmental N (context OR resource* OR stressor)) OR material resource* OR facilitat* OR barrier* OR salient event* OR opportunit* ) AND AB ( (environmental N (context OR resource* OR stressor)) OR material resource* OR facilitat* OR barrier* OR salient event* OR opportunit* ) |
| S33 | TI ( (social OR group OR collective OR shared) N (influence* OR norm* OR pressure* OR conform* OR comparison* OR identity OR conflict* OR support)) OR ((subjective OR descriptive) N3 norms) OR modelling OR observational learning ) AND AB ( (social OR group OR collective OR shared) N (influence* OR norm* OR pressure* OR conform* OR comparison* OR identity OR conflict* OR support)) OR ((subjective OR descriptive) N3 norms) OR modelling OR observational learning ) |
| S34 | TI ( emotion* OR feelings OR emotional representation* OR mood OR depress* OR fear OR anxiety OR ((positive OR negative) N affect) ) AND AB ( emotion* OR feelings OR emotional representation* OR mood OR depress* OR fear OR anxiety OR ((positive OR negative) N affect) ) |
| S35 | TI ( behavioral regulation OR behavioural regulation OR normalisation OR normalization OR self-monitoring OR self monitoring OR self-regulation OR self regulation OR symptom perception OR social messages OR illness representation* OR treatment control OR ((approach OR avoidance) N coping) ) AND AB ( behavioral regulation OR behavioural regulation OR normalisation OR normalization OR self- monitoring OR self monitoring OR self-regulation OR self regulation OR symptom perception OR social messages OR illness representation* OR treatment control OR ((approach OR avoidance) N coping) ) |
| S36 | TI ( patient* N3 (attitude* OR acceptance* OR satisfaction) ) AND AB ( patient* N3 (attitude* OR acceptance* OR satisfaction) ) |
| S37 | (MH "Attitude to Health+") OR (MH "Health Beliefs") OR (MH "Health Knowledge") |
| S38 | (MH "Attitude to Illness") |
| S39 | (MH "Patient Satisfaction") |
| S40 | (MH "Social Identity") OR (MH "Self-Efficacy") |
| S41 | (MH "Emotions+") |
| S42 | (MH "Intention") |
| S43 | (MH "Ajzen's Theory of Planned Behavior") |
| S4 | (MH "Ajzen-Fishbein Theory of Reasoned Action") |
| S45 | (MH "Attitude to Risk") |
| S46 | (MH "Self Regulation") |
| S47 | S22 OR S23 OR S24 OR S25 OR S26 OR S27 OR S28 OR S29 OR S30 OR S31 OR S32 OR S33 OR S34 OR S35 OR S36 OR S37 OR S38 OR S39 OR S40 OR S41 OR S42 OR S43 OR S44 OR S45 OR S46 |

EMBASE Classic + EMBASE (OVID interface 1953-2015 Week 44)

| Step Number | Search Terms |
| --- | --- |
| S1 | exp cerebrovascular disease/ |
| S2 | ((cva$ or stroke$ or poststroke$ or post-stroke$ or post stroke$) adj6 (people or patient$ or inpatient$ or outpatient$ or adult$ or survivor$ or victim$ or individual$ or client$ or population$ or community or subject$)).tw. |
| S3 | (cerebrovascular$ or cerebral vascular).tw |
| S4 | (cerebral or cerebellar or brain$ or vertebrobasilar).tw. |
| S5 | (infarct$ or isch?emi$ or thrombo$ or apoplexy or emboli$).tw. |
| S6 | 4 and 5 |
| S7 | (cerebral or intracerebral or intracranial or brain$ or cerebellar or subarachnoid).tw. |
| S8 | (accident$ or h?emorrhag$).tw. |
| S9 | 7 and 8 |
| S10 | 1 or 2 or 3 or 6 or 9 |
| S11 | exp patient compliance/ |
| S12 | (adhere$ or nonadhere$ or non-adhere$).tw. |
| S13 | (complian$ or noncomplian$ or non-complian$).tw. |
| S14 | ((refusal or refuse$) adj3 (medicine$ or medication$ or drug$ or prescription$ or tablet$ or pharmaceutic$)).tw. |
| S15 | ((medicine$ or medication$ or drug$ or prescription$ or tablet$ or pharmaceutic$) adj3 (dropout$ or drop-out$ or drop out$)).tw. |
| S16 | ((medicine$ or medication$ or drug$ or prescription$ or tablet$ or pharmaceutic$) adj3 (stop$ or abandon$)).tw. |
| S17 | (persist$ or concordan$ or accept$ or co-operat$ or cooperat$ or co operat$ or conform$ or medicine taking).tw. |
| S18 | (knowledg$ or awareness or understand$ or information).tw. |
| S19 | (skill$ or abilit$ or expertise or competen$ or capabilit$ or response efficacy).tw. |
| S20 | ((social adj (role or identity)) or (professional adj (role or identity or confidence)) or identity).tw. |
| S21 | ((belief$ adj2 capabilit$) or self-efficacy or self-confiden$ or perceived competence or self-esteem or empowerment or perceived behavio?ral control or self-control or self control or performance).tw. |
| S22 | (optimis$ or positiv$ or hopefulness or assurance or pessimis$ or negativ$).tw. |
| S23 | ((belief$ adj2 (consequence$ or cost$)) or (outcome adj (expectations or expectancies)) or risk perception$ or perceived benefit$ or anticipated emotion$ or necessity belief$ or concern$ or response cost$ or coping appraisal$ or perceived vulnerability or perceived likelihood).tw. |
| S24 | (reinforce$ or support$ or reward$ or incentiv$ or punish$ or consequen$).tw. |
| S25 | (intent$ or intend$ or plan$ or motivation or health motivation or pre-contemplation or contemplation or preparation or maintain$ or behaviour termination or confiden$ or temptation or consciousness raising or dramatic relief or self-reevaluation or environmental reevaluation or self-liberation or helping relationships or counterconditioning or reinforcement management or stimulus control or social lineation).tw. |
| S26 | (goal$ or action planning or target setting).tw. |
| S27 | (memory or attention or decision$ or judgement$ or cogniti$).tw. |
| S28 | ((environmental adj (context$ or resource$ or stressor$)) or material resource$ or facilitat$ or barrier$ or salient event$ or opportunit$).tw. |
| S29 | (((social or group or collective or shared) adj (Influence$ or norm$ or pressure$ or conform$ or comparison$ or identity or conflict or support)) or ((subjective or descriptive) adj norms) or modelling or observational learning).tw. |
| S30 | (emotion$ or feelings or emotional representation$ or mood or depress$ or fear or anxiety or ((positive or negative) adj affect)).tw. |
| S31 | (behavio?ral regulation or normali?ation or self-monitoring or self monitoring or self-regulation or self regulation or symptom perception or social messages or illness representation$ or treatment control or ((approach or avoidance) adj coping)).tw. |
| S32 | (patient$ adj3 (attitude$ or acceptance$ or satisfaction)).tw. |
| S33 | exp patient satisfaction/ |
| S34 | exp patient attitude/ |
| S35 | exp social status/ |
| S36 | exp self concept/ |
| S37 | exp "Theory of Planned Behavior"/ |
| S38 | exp "Theory of Reasoned Action"/ |
| S39 | exp emotion/ |
| S40 | social support/ |
| S41 | exp illness behavior/ |
| S42 | 11 or 12 or 13 or 14 or 15 or 16 or 17 |
| S43 | 18 or 19 or 20 or 21 or 22 or 23 or 24 or 25 or 26 or 27 or 28 or 29 or 30 or 31 or 32 or 33 or 34 or 35 or 36 or 37 or 38 or 39 or 40 or 41 |
| S44 | 10 and 42 and 43 |

Ovid MEDLINE(R) In-Process & Other Non-Indexed Citations and Ovid MEDLINE(R) (1953-Week 44 2015)

| Step Number | Search Terms |
| --- | --- |
| S1 | exp Cerebrovascular Disorders/ |
| S2 | (cerebrovascular$ or cerebral vascular).tw. |
| S3 | ((cva$ or stroke$ or poststroke$ or post-stroke$ or post stroke$) adj6 (people or patient$ or inpatient$ or outpatient$ or adult$ or survivor$ or victim$ or individual$ or client$ or population$ or community or subject$)).tw. |
| S4 | (cerebral or cerebellar or brain$ or vertebrobasilar).tw. |
| S5 | (infarct$ or isch?emi$ or thrombo$ or apoplexy or emboli$).tw. |
| S6 | 4 and 5 |
| S7 | (cerebral or intracerebral or intracranial or brain$ or cerebellar or subarachnoid).tw. |
| S8 | (accident$ or h?emorrhag$).tw. |
| S9 | 7 and 8 |
| S10 | 1 or 2 or 3 or 6 or 9 |
| S11 | exp Patient Compliance/ |
| S12 | exp Treatment Refusal/ |
| S13 | exp Patient Dropouts/ |
| S14 | (complian$ or noncomplian$ or non-complian$).tw. |
| S15 | (adhere$ or nonadhere$ or non-adhere$).tw. |
| S16 | ((refusal or refuse$) adj3 (medicine$ or medication$ or drug$ or prescription$ or tablet$ or pharmaceutic$)).tw. |
| S17 | ((medicine$ or medication$ or drug$ or prescription$ or tablet$ or pharmaceutic$) adj3 (dropout$ or drop-out$ or drop out$)).tw. |
| S18 | ((medicine$ or medication$ or drug$ or prescription$ or tablet$ or pharmaceutic$) adj3 (stop$ or abandon$)).tw. |
| S19 | (persist$ or concordan$ or accept$ or co-operat$ or cooperat$ or co operat$ or conform$ or medicine taking).tw. |
| S20 | 11 or 12 or 13 or 14 or 15 or 16 or 17 or 18 or 19 |
| S21 | (knowledg$ or awareness or understand$ or information).tw. |
| S22 | (skill$ or abilit$ or expertise or competen$ or capabilit$ or response efficacy).tw. |
| S23 | ((social adj (role or identity)) or (professional adj (role or identity or confidence)) or identity).tw. |
| S24 | ((belief$ adj2 capabilit$) or self-efficacy or self-confiden$ or perceived competence or self-esteem or empowerment or perceived behavio?ral control or self-control or self control or performance).tw. |
| S25 | (optimis$ or positiv$ or hopefulness or assurance or pessimis$ or negativ$).tw. |
| S26 | ((belief$ adj2 (consequence$ or cost$)) or (outcome adj (expectations or expectancies)) or risk perception$ or perceived benefit$ or anticipated emotion$ or necessity belief$ or concern$ or response cost$ or coping appraisal$ or perceived vulnerability or perceived likelihood).tw. |
| S27 | (reinforce$ or support$ or reward$ or incentiv$ or punish$ or consequen$).tw. |
| S28 | (Intent$ or intend$ or plan$ or motivation or health motivation or pre-contemplation or contemplation or preparation or maintain$ or behavio?r termination or confiden$ or temptation or consciousness raising or dramatic relief or self-reevaluation or environmental reevaluation or self-liberation or helping relationships or counterconditioning or reinforcement management or stimulous control or social lineation).tw. |
| S29 | (goal$ or action planning or target setting).tw. |
| S30 | (memory or attention or decision$ or judgement$ or cogniti$).tw. |
| S31 | ((environmental adj (context$ or resource$ or stressor$)) or material resource$ or facilitat$ or barrier$ or salient event$ or opportunit$).tw. |
| S32 | (((social or group or collective or shared) adj (influence$ or norm$ or pressure$ or conform$ or comparison$ or identity or conflict or support)) or ((subjective or descriptive) adj norms) or modelling or observational learning).tw. |
| S33 | (emotion$ or feelings or emotional representation$ or mood or depess$ or fear or anxiety or ((positive or negative) adj affect)).tw. |
| S34 | (behavio?ral regulation or normali?ation or self-monitoring or self monitoring or self-regulation or self regulation or symptom perception or social messages or illness representation$ or treatment control or ((approach or avoidance) adj coping)).tw. |
| S35 | (patient$ adj3 (attitude$ or acceptance$ or satisfaction)).tw. |
| S36 | exp Patient Satisfaction/ |
| S37 | exp Attitude to Health/ |
| S38 | exp Social Identification/ |
| S39 | exp Self Efficacy/ |
| S40 | Intention/ |
| S41 | exp Emotions/ |
| S42 | Social Support/ |
| S43 | 21 or 22 or 23 or 24 or 25 or 26 or 27 or 28 or 29 or 30 or 31 or 32 or 33 or 34 or 35 or 36 or 37 or 38 or 39 or 40 or 41 or 42 |
| S44 | 10 and 20 and 43 |

psycINFO (OVID interface 1953-November week 1 2015)

| Step Number | Search Terms |
| --- | --- |
| S1 | exp Cerebrovascular Accidents/ |
| S2 | exp Cerebrovascular Disorders/ |
| S3 | ((cva$ or stroke$ or poststroke$ or post-stroke$ or post stroke$) adj6 (people or patient$ or inpatient$ or outpatient$ or adult$ or survivor$ or victim$ or individual$ or client$ or population$ or community or subject$)).tw. |
| S4 | (cerebrovascular$ or cerebral vascular).tw. |
| S5 | (cerebral or cerebellar or brain$ or vertebrobasilar).tw. |
| S6 | (infarct$ or isch?emi$ or thrombo$ or apoplexy or emboli$).tw. |
| S7 | 5 and 6 |
| S8 | (cerebral or intracerebral or intracranial or brain$ or cerebellar or subarachnoid).tw. |
| S9 | (accident$ or h?emorrhag$).tw. |
| S10 | 8 and 9 |
| S11 | 1 or 2 or 3 or 4 or 7 or 10 |
| S12 | exp Treatment Compliance/ |
| S13 | exp Treatment Dropouts/ |
| S14 | exp Treatment Refusal/ |
| S15 | (complian$ or noncomplian$ or non-complian$).tw. |
| S16 | (adhere$ or nonadhere$ or non-adhere$).tw. |
| S17 | (persist$ or concordan$ or accept$ or co-operat$ or cooperat$ or co operat$ or conform$ or medicine taking).tw. |
| S18 | 12 or 13 or 14 or 15 or 16 or 17 |
| S19 | (knowledg$ or awareness or understand$ or information).tw. |
| S20 | (skill$ or abilit$ or expertise or competen$ or capabilit$ or response efficacy).tw. |
| S21 | ((social adj (role or identity)) or (professional adj (role or identity or confidence)) or identity).tw. |
| S22 | ((belief$ adj2 capabilit$) or self-efficacy or self-confiden$ or perceived competence or self-esteem or empowerment or perceived behavio?ral control or self-control or self control or performance).tw. |
| S23 | (optimis$ or positiv$ or hopefulness or assurance or pessimis$ or negativ$).tw. |
| S24 | ((belief$ adj2 (consequence$ or cost$)) or (outcome adj (expectations or expectancies)) or risk perception$ or perceived benefit$ or anticipated emotion$ or necessity belief$ or concern$ or response cost$ or coping appraisal$ or perceived vulnerability or perceived likelihood).tw. |
| S25 | (reinforce$ or support$ or reward$ or incentiv$ or punish$ or consequen$).tw. |
| S26 | (intent$ or intend$ or plan$ or motivation or health motivation or pre-contemplation or contemplation or preparation or maintain$ or behavio?r termination or confiden$ or temptation or consciousness raising or dramatic relief or self-reevaluation or environmental reevaluation or self-liberation or helping relationships or counterconditioning or reinforcement management or stimulus control or social lineation).tw. |
| S27 | (goal$ or action planning or target setting).tw. |
| S28 | (memory or attention or decision$ or judgement$ or cogniti$).tw. |
| S29 | ((environmental adj (context$ or resource$ or stressor$)) or material resource$ or facilitat$ or barrier$ or salient event$ or opportunit$).tw. |
| S30 | (((social or group or collective or shared) adj (Influence$ or norm$ or pressure$ or conform$ or comparison$ or identity or conflict or support)) or ((subjective or descriptive) adj norms) or modelling or observational learning).tw. |
| S31 | (emotion$ or feelings or emotional representation$ or mood or depress$ or fear or anxiety or ((positive or negative) adj affect)).tw. |
| S32 | (behavio?ral regulation or normali?ation or self-monitoring or self monitoring or self-regulation or self regulation or symptom perception or social messages or illness representation$ or treatment control or ((approach or avoidance) adj coping)).tw. |
| S33 | (patient$ adj3 (attitude$ or acceptance$ or satisfaction)).tw. |
| S34 | exp Treatment Barriers/ |
| S35 | exp Health Knowledge/ |
| S36 | exp Client Attitudes/ |
| S37 | exp Health Attitudes/ |
| S38 | Ability/ |
| S39 | exp Social Identity/ |
| S40 | exp Self Efficacy/ |
| S41 | Self Control/ |
| S42 | Intention/ |
| S43 | exp Reasoned Action/ or Planned Behavior/ |
| S44 | exp Emotional States/ |
| S45 | Risk Perception/ |
| S46 | Social Support/ |
| S47 | Self Regulation/ |
| S38 | Illness Behavior/ |
| S49 | exp Observational Learning/ |
| S50 | 19 or 20 or 21 or 22 or 23 or 24 or 25 or 26 or 27 or 28 or 29 or 30 or 31 or 32 or 33 or 34 or 35 or 36 or 37 or 38 or 39 or 40 or 41 or 42 or 43 or 44 or 45 or 46 or 47 or 48 or 49 |
| S51 | 11 and 18 and 50 |

WEB OF SCIENCE (1953-November 2015; inclusive of conference proceedings)

| Step Number | Search Terms |
| --- | --- |
| S1 | TS=((cva* or stroke* or poststroke* or post-stroke* or "post stroke*")) AND TS=((NEAR6 people or patient* or inpatient* or outpatient* or adult* or survivor* or victim* or individual* or client* or population* or community or subject*)) |
| S2 | TS=((cerebrovascular* or "cerebral vascular")) |
| S3 | TOPIC: ((cerebral or cerebellar or brain* or vertebrobasilar)) |
| S4 | TS=((infarct* or ischemi* or ischaemi* or thrombo* or apoplexy or emboli*)) |
| S5 | #4 AND #3 |
| S6 | TOPIC: ((cerebral or intracerebral or intracranial or brain* or cerebellar or subarachnoid)) |
| S7 | TS=((accident* or hemorrhag* or haemorrhag*)) |
| S8 | #7 AND #6 |
| S9 | #8 OR #5 OR #2 OR #1 |
| S10 | TOPIC: ((complian* or noncomplian* or non-complian*)) |
| S11 | TS=((adhere* or nonadhere* or non-adhere*)) |
| S12 | TS=((persist* or concordan* or accept* or co-operat* or cooperat* or "co operat*" or conform* or "medicine taking")) |
| S13 | TOPIC: ((refusal or refuse*)) *AND* TOPIC: ((NEAR3 medicine* or medication* or drug* or prescription* or tablet* or pharmaceutic*)) |
| S14 | TS=((medicine* or medication* or drug* or prescription* or tablet* or pharmaceutic*)) AND TS=((NEAR3 dropout* or drop-out* or "drop out*")) |
| S15 | TOPIC: ((medicine* or medication* or drug* or prescription* or tablet* or pharmaceutic*)) *AND* TOPIC: (( NEAR3 stop* or abandon*)) |
| S16 | #15 OR #14 OR #13 OR #12 OR #11 OR #10 |
| S17 | TOPIC: ((knowledg* or awareness or understand* or information)) |
| S18 | TS=((skill* or abilit* or expertise or competen* or capabilit* or "response efficacy")) |
| S19 | TOPIC: (((Social NEAR (role or identity)) or (professional NEAR (role or identity or confidence)) or identity)) |
| S20 | TS=(((belief* NEAR2 capabilit*) or self-efficacy or self-confiden* or "perceived competence" or self-esteem or empowerment or "perceived behavioral control" or "perceived behavioural control" or self-control or "self control" or performance)) |
| S21 | TOPIC: ((optimis* or positiv* or hopefulness or assurance or pessimis* or negativ*)) |
| S22 | TS=(((belief* NEAR2 (consequence* or cost*)) or "outcome expectations" or "outcome expectancies" or "risk perception*" or "perceived benefit*" or "anticipated emotion*" or "necessity belief*" or concern* or "response cost*" or "coping appraisal*" or "perceived vulnerability" or "perceived likelihood")) |
| S23 | TOPIC: ((reinforce* or support* or reward* or incentiv* or punish* or consequen*)) |
| S24 | TS=((Intent* or intend* or plan* or motivation or "health motivation" or pre-contemplation or contemplation or preparation or maintain* or "behavior termination" or “behaviour termination” or confiden* or temptation or "consciousness raising" or "dramatic relief" or self-reevaluation or "environmental reevaluation" or self-liberation or "helping relationships" or counterconditioning or "reinforcement management" or "stimulus control" or "social lineation")) |
| S25 | TS=((goal* or "action planning" or "target setting")) |
| S26 | TOPIC: ((memory or attention or decision* or judgement* or cogniti*)) |
| S27 | TS=(((environmental NEAR (context or resource* or stressor*)) or "material resource*" or facilitat* or barrier* or "salient event*" or opportunit*)) |
| S28 | TS=(((social or group or collective or shared) NEAR (Influence* or norm* or pressure* or conform* or comparison* or identity or conflict or support) or ((subjective or descriptive) NEAR norms) or modelling or "observational learning")) |
| S29 | TS=((emotion* or feelings or "emotional representation*" or mood or depress* or fear or anxiety or ((positive or negative) NEAR affect))) |
| S30 | TS=(("behavioral regulation" or “behavioural regulation” or normalisation or normalization or self-monitoring or "self monitoring" or self-regualtion or "self regulation" or "symptom perception" or "social messages" or "illness representation*" or "treatment control" or ((approach or avoidance) NEAR coping))) |
| S31 | TOPIC: ((patient*)) *AND* TOPIC: ((NEAR3 attitude* or acceptance* or satisfaction)) |
| S32 | #31 OR #30 OR #29 OR #28 OR #27 OR #26 OR #25 OR #24 OR #23 OR #22 OR #21 OR #20 OR #19 OR #18 OR #17 |
| S33 | (#32 AND #16 AND #9) |
