# Supplementary material for: Psychological Determinants of Medication Adherence in Stroke Survivors: a Systematic Review of Observational Studies
Source: Ann Behav Med. 2017 Apr 18;51(6):833–45. doi: 10.1007/s12160-017-9906-0 (PMC5636868; doi:10.1007/s12160-017-9906-0)
Supplement: Supplementary file 2 — (DOCX 57.8 kb) [file 12160_2017_9906_MOESM2_ESM.docx]

Supplement Table 2. Methods of medication adherence measurement

| Outcome Measure | Bushnell (2010) | Bushnell (2011) | Coetzee (2008) | Edmondson (2013) | Glader (2010) | Kronish (2012) | Kronish (2013) | O’Carroll (2011) | Phillips (2014) | Phillips (2015) | Sjolander (2011) | Sjolander (2013) |
| --- | --- | --- | --- | --- | --- | --- | --- | --- | --- | --- | --- | --- |
| Eight-item Morisky | + | + |  | + |  | + | + |  | + | + |  |  |
| MARS |  |  |  |  |  |  |  | + |  |  |  | + |
| TAS |  |  | + |  |  |  |  |  |  |  |  |  |
| Record Comparison/ Linkage | + | + |  |  | + |  |  |  |  |  |  |  |
| Prescription Refills |  |  |  |  |  |  |  |  |  |  | + |  |
| Urine Sample |  |  |  |  |  |  |  | + |  |  |  |  |
| Pill counts |  |  | + |  |  |  |  |  |  |  |  |  |

MARS- Medication Adherence Report Scale; TAS- Treatment Adherence Scale
