# Supplementary material for: Psychological Determinants of Medication Adherence in Stroke Survivors: a Systematic Review of Observational Studies
Source: Ann Behav Med. 2017 Apr 18;51(6):833–45. doi: 10.1007/s12160-017-9906-0 (PMC5636868; doi:10.1007/s12160-017-9906-0)
Supplement: Supplementary file 3 — (DOCX 95 kb) [file 12160_2017_9906_MOESM3_ESM.docx]

Supplement Table 3. Quality Assessment Checklist

| Study (first author and year) | Explicit *a priori* aims | Definition/ size of population under investigation | Sample size calculation | Justification that sample is representative of population | Inclusion/ exclusion criteria stated | Demographic details | Research independent of routine care/ practice | Justification of validity/ reliability of measures | Original questionnaire available | Response/ drop-out rate specified | Justification of response/ drop-out rate | Discussion of generalisability | Statement of source of funding | Marks lost | Percentage of maximum quality score |
| --- | --- | --- | --- | --- | --- | --- | --- | --- | --- | --- | --- | --- | --- | --- | --- |
| Kronish (2012) | + | + | - | + | + | + | + | + | + | N/A | N/A | + | + | 1 | 90.9 |
| Kronish (2013) | + | + | - | + | + | + | + | + | + | N/A | N/A | + | + | 1 | 90.9 |
| Edmondson (2013) | + | + | - | + | + | + | + | + | + | N/A | N/A | + | + | 1 | 90.9 |
| Coetzee (2008) | + | + | - | - | + | + | + | + | + | + | + | + | + | 2 | 84.6 |
| Sjolander (2013) | + | + | + | + | + | + | + | + | + | + | - | - | + | 2 | 84.6 |
|  |  |  |  |  |  |  |  |  |  |  |  |  |  |  |  |
| O’Carroll (2011) | + | + | + | - | + | - | + | + | - | + | + | + | + | 3 | 76.9 |
|  |  |  |  |  |  |  |  |  |  |  |  |  |  |  |  |
| Phillips (2014) | + | + | - | + | + | + | + | + | + | N/A | N/A | - | + | 3 | 72.7 |
| Phillips (2015) | + | + | - | + | + | + | + | + | + | N/A | N/A | - | - | 3 | 72.7 |
|  |  |  |  |  |  |  |  |  |  |  |  |  |  |  |  |
| Glader (2010) | + | + | - | + | + | + | + | - | + | + | - | - | + | 4 | 69.2 |
| Sjolander (2011) | + | + | - | + | + | + | + | - | + | + | - | - | + | 4 | 69.2 |
| Bushnell (2010) | + | + | - | - | + | + | + | - | - | + | - | + | + | 5 | 61.5 |
| Bushnell (2011) | + | + | - | - | + | + | + | - | - | + | - | + | + | 5 | 61.5 |
|  |  |  |  |  |  |  |  |  |  |  |  |  |  |  |  |
| **Total** | 12/12 | 12/12 | 2/12 | 8/12 | 12/12 | 11/12 | 12/12 | 8/12 | 9/12 | 7/7 | 2/7 | 7/12 | 11/12 | **Mean**  2.8 | **Mean**  77.1 |
